# Oral health promotion practices: a survey of Florida child care center directors

**DOI:** 10.1186/s12903-018-0562-y

**Published:** 2018-06-01

**Authors:** Vinodh Bhoopathi, Ajay Joshi, Romer Ocanto, Robin J. Jacobs

**Affiliations:** 1 Department of Pediatric Dentistry and Community Oral Health Sciences, Temple University Maurice H. Kornberg School of Dentistry, 3223 N Broad Street, Philadelphia, PA 19140 USA; 2 Pediatric Dentistry Department, Indiana University School of Dentistry, 1121 W. Michigan Street, Indianapolis, IN 46202 USA; 3 Department of Pediatric Dentistry, Nova Southeastern University College of Dental Medicine, 3200 S University Drive, Fort Lauderdale, FL 33328 USA; 4 Department of Family and Community Medicine – Research Programs, Baylor College of Medicine, 3701 Kirby Drive, Suite 600, Houston, TX 77098 USA

**Keywords:** Oral health, Health promotion, Day care, Child care centers, Dental caries, Prevention, Head start, Early head start

## Abstract

**Background:**

To understand the oral health promotion practices (OHPPs) in Florida licensed childcare centers (CCCs), we surveyed the childcare center directors (CCCDs) employed at these centers. We determined if CCC’s affiliation with Early Head Start/Head Start (EHS/HS) programs was associated with the number of OHPPs implemented.

**Methods:**

For this cross-sectional study we emailed a pretested 45-item online survey to unduplicated email addresses of 5142 licensed CCCDs as listed in the publicly available Florida Department of Child and Family services database. Univariate and bivariate analyses were conducted. In addition, a Poisson regression model predicting higher numbers of OHPPs implemented was conducted.

**Results:**

A response rate of 19.4% was estimated. CCCDs reporting to implement a higher number of OHPPs in their CCCs were more likely to have longer work experience (b = 0.006, 95% CI: 0.001,0.012 *p* = 0.03), work in EHS/HS affiliated centers (b = 0.7, 95%CI: 0.48,0.91) *p* < 0.001), and have more positive attitudes about pediatric oral health (b = 0.08, 95%CI: 0.05, 0.10) *p* < 0.001). CCCDs with more self-perceived barriers reported implementing a lower number of OHPPs (b = − 0.046, 95% CI: -0.09, − 0.003 *p* = 0.035) compared to their counterparts.

**Conclusions:**

A significant association between a CCC’s affiliation with EHS/HS programs and the number of OHPPs implemented was observed. In addition, CCCD’s years of experience, attitudes towards oral health, and self-perceived barriers in implementing OHPPs were also associated with the number of OHPPs implemented.

## Background

The number of child care facilities in the U.S. rose from 262,511 in 1987 to 766,401 in 2007, indicating an increasing trend in the establishment of such facilities [1]. There were 32.7 million children in ‘out-of-home’ child care facilities in year 2011, of which most (20.2 million) were aged 5–14 years; while the remaining 12.5 million were aged 0 to 4 years [2]. Preschoolers of employed and non-employed mothers spent approximately 36 h and 21 h respectively per week in these facilities [2]. Because a significant proportion of children spend so much time in these facilities, health intervention and promotion programs can be implemented in these settings to promote the health of the enrolled children.

One significant public health problem is an ongoing epidemic of dental caries in the U.S. children. The 2011–2012 National Health and Nutrition Examination Survey data showed that at least 40% of 2 to 8 year old children experienced dental caries in their primary teeth, with at least 14% having untreated tooth decay, suggesting that despite needing dental care, it was not received [3]. Approximately 21% of children ages 6 to 11, and 53% of adolescents aged 12 to 19 years had experienced dental caries [3]. This national data suggests that children develop dental caries all through their childhood.

Since many children spend a portion of their day in CCCs, centers provide an ideal setting to adopt measures to prevent dental caries, especially since most children enrolled in CCCs fall into the susceptible age range for dental caries. CCCs and childcare center directors (CCCDs) could take an active role to prevent dental diseases and promote oral health of all children enrolled in these centers by educating children and their parents about the importance of maintaining proper oral health, and adopting good oral health promotion practices (OHPPs) [4].

The American Academy of Pediatric Dentistry (AAPD) recognizes the importance and impact of oral health promotion within CCCs, based on children’s increased utilization of and time spent in these facilities for daily care [5]. The AAPD released a set of oral health guidelines addressing dental disease prevention and oral health promotion in out-of-home child care settings targeting CCCs, pediatric dentists, other health care professionals, legislators and policy makers [5]. This policy encourages CCCs to implement oral health promotion practices (OHPPs) to reduce a child’s risk of acquiring early childhood caries and the risk of dental trauma within their centers.

Very few studies have assessed the oral health related policies and regulations in daycare or childcare centers in the U.S. [6–8]. Little is known about licensed CCCs in the state of Florida, and the type of OHPPs implemented within these centers. Florida CCCs provide a unique opportunity to explore oral health promotion practices because children in Florida experience poorer oral health and lack adequate dental care access compared to children in many other states [9]. Therefore our study surveyed child care center directors (CCCDs) employed in Florida licensed CCCs to determine which of the 8 selected AAPD recommended OHPPs were already implemented, and the factors associated with a higher number of OHPPs implemented. Because evidence [10] shows that children in CCCs affiliated with Head Start [HS] programs are significantly more likely to receive health care screenings and consultations compared to non-HS programs, we tested if there was any association between number of OHPPs implemented and the CCC’s affiliation with Early Head Start/Head start (EHS/HS) programs. EHS/HS programs are federal programs that promote school readiness among low-income children 0 to 5 years of age. These programs offer comprehensive early child hood education, health care services, nutrition, and parental involvement services. Many EHS/HS programs are based in preschools, and others are located in licensed childcare centers or family childcare homes.

## Methods

### Study sample

This cross sectional study was approved by the Nova Southeastern University Health Professions Division Institution Review Board (IRB) (Protocol number: CGG2013–19). The target population for this study was CCCDs working in licensed CCCs within the State of Florida. A publicly available database comprising of unduplicated names and email address of Florida CCCDs (*n* = 5142) was retrieved from the Florida Department of Children and Families website in January 2014. Eight hundred and seventy seven CCCDs responded, 53 opted out, and 631 email addresses were invalid. The overall survey response rate was estimated at 19.4% (877/4511).

### Survey instrument

The authors developed the 45-item survey by adapting questions from previously tested and validated surveys [11–13]. AAPD oral health policies for CCCs [5] were also used to construct questions to assess OHPPs implemented in the CCCs. A group of five pediatric dentists provided detailed feedback on the structure and content of the first draft of the survey. The second draft of the modified survey was pretested with 10 CCCDs in Broward county, Florida**.** The survey was pilot tested through cognitive interviews using the concurrent think aloud method with probes [14]. These procedures we believe improved the content and face validity of the survey.

### Data collection

The pilot tested survey was uploaded on the Survey Monkey® online platform (www.surveymonkey.com). We used Dillman’s guidelines such as: 1) repeated contacts, 2) varying messages across reminders, 3) caution to minimize spam, and 4) testing the compatibility of the online surveys on different devices and softwares, to contact the CCCDs and boost the responses. [15]. For repeated contact, we included: (1) an introductory email informing the CCCDs about the upcoming survey; (2) an email with a message about the intent of the survey, why they were selected to be part of the study, and the importance of their participation; and (3) reminder emails, sent every 2 weeks intervals (a total of 3 reminders), on early Monday morning hours with personalized links, to both partial and non-respondents over a 6-week period. We varied the content of the email message with all reminders to vary the stimulus across email contacts. To minimize the likelihood of the online survey being flagged as spam we used plain text messages, instead of HTML messages. And finally, we tested the online survey on iphones, androids, desktops, and different software and hard ware configurations. The online version of the survey was also tested for operational and typological issues. The survey was initially sent to the sample in January 2014, and was kept open until the end of March 2014.

#### Independent variables

##### Demographic variables

Questions were asked about (but not limited to) CCCDs age, gender, race, ethnicity, highest form of education completed, annual income, years of experience as a CCCD, and if they had a child of their own.

##### Pediatric oral health knowledge (knowledge)

Three questions/statements assessing the CCCD’s knowledge about pediatric oral health, were adapted from a previous study [11]. The first statement specified that the parents should start cleaning a child’s mouth at the age of 1 (True or False response). The correct answer to this question was False, because cleaning children’s teeth should begin as soon as the first tooth erupts. The second True/False statement indicated that a child’s first dental visit should be at 2 years. The correct answer is False because children should have a first dental office visit at the age of 1. The third statement asked the respondents to correctly choose the most common chronic childhood disease for children younger than 7 years old from four possible responses (Asthma, Hay Fever, Tooth decay, and Chicken Pox). The correct answer for this question was tooth decay. Correct answers were assigned a score of 1 and were summed to create a composite knowledge score (range 0 to 3). Higher composite scores indicated that CCCDs had a higher level of pediatric oral health knowledge.

##### Attitudes towards pediatric oral health (attitudes)

A 5-point Likert scale (Strongly Agree to Strongly Disagree; coded as 1 to 5) was used to rate the following attitude-based statements: 1) Cleaning baby teeth is not important because they fall out anyway; 2) My center has too many activities to devote any time to dental health; 3) Teaching children younger than 3 years about dental health is too difficult; and 4) I don’t believe that the activities that we provide in the center will prevent cavities [12]. A composite attitude score (range 0 to 20) was derived by summing the answers with higher scores indicating positive attitudes towards promoting children’s oral health. An acceptable internal consistency (Cronbach’s alpha = 0.706) was estimated for the likert scales measuring attitudes.

##### Self-perceived barriers (barriers)

Possible barriers to implementing OHPPs were listed with a check box option. CCCDs could check any of the items that apply. The list of barriers were: 1) Insufficient funding to promote pediatric oral health; 2) Parents’ negative attitudes towards child safety and oral health; 3) Parental cultural/religious barriers; 4) Parents’ language barriers; 5) Insufficient training of center staff about oral health promotion topics; 6) Insufficient space to implement OHPPs; 7) Inadequate time to implement OHPPs; 8) Infection control concerns; and 9) other (open response). All checked responses (coded as 1) were summed together to derive a composite SPB score (ranging from 0 to 9), with higher scores indicating that CCCDs had greater difficulty implementing OHPPs in their centers.

##### Affiliation with EHS/HS programs (main independent variable)

CCCDs were asked using a check box option to choose if their center was affiliated with EHS/HS programs or not. A checked response meant that CCCD was at a center affiliated with EHS/HS programs.

#### Main outcome variable

##### Oral health promotion practices (OHPPs)

CCCs implementation of OHPPs, as recommended by the AAPD’s “Policy on Oral Health in Child Care Centers” [5] was measured by asking 8 binary option (yes/ no) questions. In order to accommodate the time constraints and to prevent potential overlap between OHPPs, researchers developed questions for only 8 out of a possible 14 AAPD recommended OHPPs. The decision to include only 8 of the 14 AAPD recommended OHPPs was made based on the feedback received from 5 pediatric dentists who provided feedback on the content and structure of the survey. The questions asked the CCCDs whether the center he/she was employed at: 1) had an oral health consultant; 2) regularly maintained dental records for enrolled children; 3) had training or educational programs for staff about traumatic dental injuries 4) had an onsite dental emergency manual; 5) regularly distributed oral health promotion materials to parents; 6) provided optimally fluoridated water for the children; 7) promoted the dental home concept to parents; and 8) encouraged children to brush their teeth after meals or snacks. All “yes” responses were considered positive responses, and were given a score of 1, while “no” responses were coded as 0. The responses were summed to derive a composite OHPS score (Score range: 0 to 8) with higher scores indicating more OHPS implemented by CCCs.

### Analyses

Data analyses were performed using the version 9.3 of the SAS statistical analysis software (SAS Institute, Inc. Cary, N.C.). Alpha coefficients were performed to test reliability between items included in the attitude-based questions. We conducted descriptive statistics to understand the characteristics of the study sample. The following variables were described through frequencies and percentages: CCCD’s age, gender, ethnicity, race, education, annual income, having a child of their own (being a parent), and the center’s affiliation with EHS/HS programs. The following variables were described through means and standard deviation: CCCD’s age, years of experience working at a CCC, knowledge, attitudes, barriers, and the self-reported number of OHPPs implemented in their center. Bivariate comparisons were conducted using chi-square tests and independent student t-tests to understand differences in the proportion of CCCDs reporting OHPPs implementation, and the overall number of OHPPs implemented in CCCs. One Poisson regression model was created which predicted the number of OHPPs implemented in Florida CCCs. We included all independent variables explained above as covariates. Multi-collinearity diagnostic analysis was performed to assess collinearity between the predictor variables that were included in the regression model, and none was detected. To assess the fit of the poisson regression model, we used the goodness-of-fit chi-squared test.

## Results

The mean age of the CCCD respondents was 48.5 ± 10.5 years and they had mean years of experience of 11.6 ± 9.3 years. A majority of the study participants were women (96%) and belonged to the White race (74%). Approximately 19% of the sample was Hispanics. The majority (65%) reported having a college degree or higher. More than 60% reported earning an annual income of less than $50,000, with just over 20% reporting an income of $50,000 and above. Only 5% of the responding CCCDs reported that their center was affiliated with EHS/HS programs.

On average, participants answered only one knowledge question out of 3 correctly [Knowledge score: 1.3 ± 0.8 (mean ± SD)]. When asked if age 1 was the correct age to initiate cleaning a child’s teeth, only 1 in 5 correctly answered “False”. Only 2 in 5 CCCDs correctly answered that the child’s first dental visit should not be at 2 years. However, an overwhelming 85% of the respondents correctly identified that tooth decay or cavities is the most common childhood disease.

The mean attitude score (16.8 ± 2.7) suggested that CCCDs had positive attitudes towards pediatric oral health. Most of the respondents (94%) believed that cleaning baby teeth was very important. Only 9% felt that that there were too many activities at the center to devote any time to children’s dental health. Most (87%) felt that teaching children younger than 3 about the importance of oral health was not difficult. More than 65% believed that providing oral health promotion activities in CCCs will prevent dental caries.

CCCDs did not perceive that there were too many barriers to implementing OHPPs in their centers (mean SPB score: 1.55 ± 1.64). Funding issues (38.5%) and lack of oral health promotion training for staff (32.7%) were the most frequently reported self-perceived barriers by CCCDs. Less frequent barriers were lack of time to address oral health (24.7%), infection control issues (15.2%), lack of space to promote adequate oral health (14.1%), and negative parental attitudes (11.6%). Few CCCDs perceived parent’s language barriers (6.6%), cultural issues (5.4%), or other issues (2.5%) to be significant barriers to providing OHPPs in their center.

**Fig. 1:**
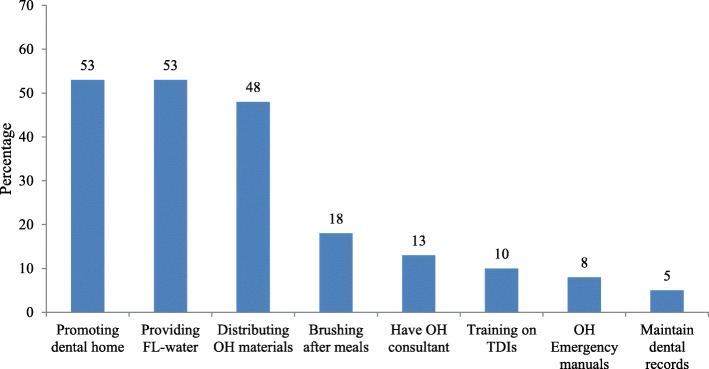
Percentage of childcare directors reporting implementation of certain oral health promotion practices in their centers

Figure 1 illustrates the percentage of respondents reporting about the implementation of 8 OHPPs in their centers. Slightly more than half of CCCDs reported that they promote the dental home concept to parents (53%) and provide optimally fluoridated water to children (53%), while the least implemented OHPPS were having an oral health emergency manual on site (8%) and maintaining children’s dental records (5%). On average, CCCDs reported implementing only 2.1 ± 1.6 (mean ± SD) out of 8 possible AAPD recommended OHPPs in their CCCs.

### Bivariate analysis

Tables 1 compares the differences in number of OHPPs implemented by selected characteristics of CCCDs. No significant differences in OHPPs implemented were observed by ethnicity, income, and having a child of their own. Male CCCDs reported a significantly higher number of OHPPs implemented compared to female CCCDs (*p =* 0.02*)*. Those belonging to a non-White racial background (*p =* 0.001*),* and those with a college degree (*p =* 0.03*)* and above reported implementing a significantly higher number of OHPPs compared to their counterparts. Table 2 compares CCCs affiliated with EHS/HS programs to unaffiliated centers. More EHS/HS affiliated CCCDs consistently reported implementing 7 OHPPs compared to their counterparts, with the exception of one OHPP. Directors in EHS/HS affiliated centers were as likely (52%) to report providing clean optimally fluoridated water throughout the day as directors in centers that are not affiliated (47%). Overall, the directors in centers affiliated with EHS/HS programs reported to have implemented a significantly higher mean number (5.1 ± 2.3) of OHPPs compared to those in centers not affiliated (1.9 ± 1.8).

**Table 1:** Mean differences in OHPPs implemented by selected CCCD characteristics

Variable	OHPPs (mean ± SD)	*p*-value
Gender		
Male	2.79 ± 2.2	*0.02*
Female	2.04 ± 1.6	
Race		
White	1.95 ± 1.5	*0.001*
Non-White	2.39 ± 1.9	
Ethnicity		
Hispanics	2.24 ± 1.7	0.15
Non-Hispanics	2.02 ± 1.6	
Income		
> =50,000	2.04 ± 1.5	0.92
< 50,000	2.03 ± 1.6	
Education		
College degree and above	2.16 ± 1.7	*0.03*
< College degree	1.89 ± 1.4	
Have Child Of your own		
Yes	2.04 ± 1.6	0.9
No	2.06 ± 1.7

**Table 2:** Factors predicting higher number of oral health promotion practices implemented in Florida child care centers

Variable	Paramester estimate	95% CI	p-value
Age	0.006	(−0.001, 0.013)	0.95
Years of experience (Higher number)	0.006	(0.0006, 0.012)	*0.03*
Gender (Male versus Female)	0.093	(−0.224, 0.41)	0.57
Race (White versus Non-Whites)	−0.08	(−0.218, 0.057)	0.25
Have a Child of your own (Yes Versus No)	0.074	(−0.073, 0.22)	0.33
Income (< 50,000 versus > = 50,000)	−0.009	(−0.15, 0.132)	0.90
Education (college degree and above versus Less than college degree)	0.033	(−0.103, 0.17)	0.64
Type of Center (Early Head Start Versus Non-Early Head Start)	0.7	(0.48, 0.914)	*<.0001*
Oral Health Knowledge (Higher number)	0.001	(−0.08, 0.08)	0.98
Attitudes (Higher number)	0.08	(0.05, 0.103)	*<.0001*
Barriers (Higher number)	−0.046	(− 0.09, − 0.003)	*0.035*

### Poisson regression analysis

The adjusted Poisson regression model predicting higher number of OHPPs implemented in Florida licensed CCCs is shown in Table 2. CCCDs employed at a center affiliated with EHS/HS programs reported implementing a higher number of OHPPs compared to CCCDs at centers not affiliated with EHS/HS programs (b = 0.7, 95%CI: 0.48,0.91) *p* < 0.001). The results also confirmed that CCCDs reporting higher number of OHPPs implemented in their centers were more likely to have longer work experience (b = 0.006, 95% CI:0.001, 0.012 *p* = 0.03), and have more positive attitudes about pediatric oral health (b = 0.08, 95%CI: 0.05, 0.10) *p* < 0.001). CCCDs who had more self-perceived barriers in implementing OHPPs reported that their centers had implemented significantly lower number of OHPPs (b = − 0.046, 95% CI: -0.09, − 0.003 *p* = 0.035). The goodness of fit test proved that the Poisson regression model fit the data reasonably well because the test was not statistically significant (*p* = 0.094).

## Discussion

Understanding the oral health promotion practices in Florida licensed CCCs is important because these centers can be utilized as alternate non-traditional settings to promote optimal oral health of children. So we conducted a survey of CCCDs in Florida licensed CCCs to examine whether their center implemented any OHPPs, and if their center’s affiliation with EHS/HS programs affected the number of OHPPs implemented.

Of the 8 OHPPs assessed, our findings indicate that, on average, CCCDs reported implementing very few OHPPs in their centers, suggesting that OHPPs may not adequately practiced in these centers. More than 80% of the CCCDs reported that their enrollees did not brush after meals, their center lacked an oral health consultant and oral health emergency manuals, the staff were not trained in traumatic dental injuries, and did not maintain children’s dental records. This indicates that, based on the CCCDs’ reports, AAPD recommended oral health prevention and promotion activities were not frequently practiced in licensed Florida CCCs. In fact, a substantial number of children younger than 5 years old were enrolled in these centers at the time of our study (more than 80%), which is problematic because this age group has high dental caries risk and oral health promotion should already be initiated. At least in this study, we did not find any association between CCCD’s oral health knowledge and the number of OHPPs implemented. However, possessing correct oral health knowledge and high oral health literacy is important for CCCDs to practice appropriate OHPPs not only for themselves but also to implement into that childcare system that will benefit the enrolled children. Therefore Florida CCCDs need more education about the importance of implementing OHPPs within their CCCs, along with the long-term impact it can have on a child’s overall health and well-being. However, it was encouraging to find that most participants (67%) reported that they might implement OHPPs in the upcoming year.

To test our hypothesis and to determine factors associated with more implemented OHPPs in Florida CCCs, we conducted an adjusted Poisson regression model that yielded interesting results. Our study found that CCCDs working in EHS/HS affiliated centers implemented more OHPPs compared to their counterparts (*p* < 0.001). We conclude that there was a significant association between the number of OHPPs implemented and the center’s affiliation with EHS/HS programs. Literature supporting this result exists, with HS centers promoting health considerably more frequently than non-HS centers [10]. In a multi-state survey a higher proportion of responding CCCDs in HS centers reported consulting health professionals and screening for health problems in enrolled children, compared to their counterparts. This is due to greater awareness about pediatric health, and CCCDs in HS centers may attach greater importance to children’s health issues [10]. More experienced CCCDs may have been more confident and efficacious compared to inexperienced CCCDs, and therefore may have implemented more OHPPs. CCCDs with positive pediatric oral health promotion attitudes were more likely to report implementing OHPPs in their centers. Evidence suggests that those with more positive attitudes about health maintenance are more likely to adopt and practice healthy behaviors for their own well-being [16]. Prior research supports the idea that more barriers (perceived and real) impede prevention program implementation [17]. The most frequently reported barriers to implementing OHPPs by study participants were: 1) insufficient funding to implement oral health programs, 2) insufficient staff training about oral health promotion, and 3) insufficient staff time for pediatric oral health promotion. Previous literature has shown that these three elements are critical to the success of any health promotion or disease prevention programs in CCCs. Therefore we recommend that CCCDs identify strategies to overcome these three barriers to health promotion. Additional open-ended responses provided insights into other potential barriers faced by CCCDs when implementing OHPPs including: dentists rarely treating and educating children younger than 3 years, few community dentists, and there is no need to enforce oral health promotion at the center because it is not required for Florida’s licensure.

Ours is the first study to survey CCCDs in Florida on relevant OHPPs in their centers. Therefore our study highlights for the first time, the status of licensed CCCs in Florida and the lack of adequate oral health promotion in these settings. Limitations of this study include but are not limited to low response rate, use of a convenience sample, and induced bias due to selective participation. Therefore our study results should be interpreted with caution. Only 5% of the respondents reported working in EHS/HS affiliated centers compared to their counterparts (95%). A previous study showed very similar findings, with only 10% of the responding CCCDs reporting to work at HS affiliated centers [10]. We did not find information about the proportion of licensed CCCs in Florida that were affiliated with EHS/HS programs and therefore we were unable to determine if non-EHS/HS CCCDs were more or less likely to participate in the survey compared to their counterparts. A very small proportion of the respondents were males. Other types of childcare facilities, such as non-licensed CCCs, group childcare homes or family child care homes, were not explored because we did not have access to these facilities. Understanding the demographic differences between respondents and non-respondents could not be accomplished because the researchers did not manually track the participants as deemed by the IRB guidelines. Due to the limited funds to execute this study, postal surveys were not economically feasible.

Because the EHS/HS affiliated centers implement more OHPPs, we believe that EHS/HS programs may serve as a model that can be integrated into non-EHS/HS affiliated programs. Because many children receive daycare in CCCs, it is imperative that policy makers and State Departments of Health focus on policies and regulations that will improve the integration of OHPPs into these settings. For example, in the State of Florida, the child care licensing program is a component of the services provided by the Department of Children and Families. This program through regulations and consultation ensures that licensing requirements are met by the childcare facilities thus preventing operation of substandard childcare programs. Such departments can add mandatory regulations related to maintaining certain oral health standards in CCCs. By doing so, optimal oral health in children can be achieved by all CCCs. Child care centers are non-traditional alternate settings where new disease prevention and health promotion programs can be implemented to improve the health of enrolled children. These settings are excellent resources to apply oral health intervention programs, provided there are few barriers.

## Conclusions

We conclude that affiliation with EHS/HS programs is associated with the number of OHPPs implemented licensed Florida licensed CCCs. In addition, CCCDs years of experience, attitudes towards oral health, and self-perceived barriers in implementing OHPPs were also associated with number of OHPPs implemented.

## Abbreviations

AAPD: American Academy of Pediatric Dentistry
CCCDs: Child care center directors
CCCs: Child care centers
EHS/HS: Early Head Start/ Head Start
HS: Head Start
IRB: Institution Review Board
OHPPs: Oral health promotion practices
